# Effects of essential oil of *Satureja khuzestanica* on the oxidative stress in experimental hyperthyroid male rat

**Published:** 2015-09-15

**Authors:** Raheleh Assaei, Zohreh Mostafavi-Pour, Naser Pajouhi, Gholam Hossein Ranjbar Omrani, Masood Sepehrimanesh, Fatemeh Zal

**Affiliations:** 1 *Endocrine and Metabolism Research Center, Shiraz University of Medical Sciences, Shiraz, Iran;*; 2 *Razi Herbal Medicines Research Center, Lorestan University of Medical Sciences, Khorramabad, Iran; *; 3 *Maternal-Fetal Medicine Research Center, Shiraz University of Medical Sciences, Shiraz, Iran; *; 4 *Recombinant Protein Laboratory, School of Advanced Medical Sciences and Technologies, Shiraz University of Medical Sciences, Shiraz, Iran;*; 5 *Department of Physiology, School of Medicine, Shiraz University of Medical Sciences, Shiraz, Iran; *; 6 *Gastroenterohepatology Research Center, Shiraz University of Medical Sciences, Shiraz, Iran; *; 7 *Reproductive Biology Department, School of Advanced Medical Sciences and Technologies, Shiraz, Iran; *; 8 *Infertility Research Center, Shiraz University of Medical Sciences, Shiraz, Iran.*

**Keywords:** Essential oil, Experimental hyperthyroidism, Glutathione, Malondialdehyde, * Satureja*

## Abstract

This work analyzes the effects of *Satureja khuzestanica* essential oil (SKEO) on the thyroid and antioxidant system, assessed by measuring levels of tri-iodothyronine (T3), thyroxine (T4), thyroid-stimulating hormone (TSH), malondialdehyde (MDA), reduced glutathione (GSH), and glutathione peroxidase (GPx) activity. Forty adult male Sprague Dawley rats (225 ± 25 g) were divided into five equal groups: one control and four hyperthyroid groups that received placebo, 200 mg kg^-1^ body weight of vitamin (Vit.) E, 225 mg kg^-1 ^body weight of SKEO, 200 and 225 mg kg^-1^ body weight of Vit. E and SKEO together, respectively. Hyperthyroidism was induced by administering of L-thyroxin in drinking water. After 30 days of L-thyroxin consumption, serum T_3_ and T_4_ levels, TSH, and oxidative stress indices were determined. Significant increase in serum T_3_, T_4_ and MDA concentrations with a simultaneous significant decrease in TSH, GSH level and GPx activity were observed in hyperthyroid group (*p* <0.05). In the treatment groups, SKEO and/or Vit. E can compensate serum MDA elevation and GPx activity reduction. Only, SKEO + Vit. E could compensate the decline of GSH levels in response to hyperthyroidism. Supplementation of SKEO, plus Vit. E as antioxidants is useful in attenuating lipid peroxidation and may potentially benefit hyperthyroid patients.

## Introduction

Thyroid hormones have a significant role in regulation of metabolism and increasing the concentration of these hormones in hyperthyroid patients increases the meta-bolism and generates the reactive oxygen species (ROS). These molecules can cause oxidative stress resulting reduced global efficacy of the antioxidant defense system.^1^ In other words, increased thyroid hormone concentrations affect the oxidant/antioxidant equilibrium and may injure the cells,^2^ which in the long time, lead to oxidative damage of many organs including the cardiovascular, respiratory and nervous system.^3^ Treatment with anti-thyroid drugs like propylthiouracil (PTU) cannot restore the balance of the disturbed oxidative and antioxidative activities.^4^^,^^5^ Thus, controlling the concentration of thyroid hormones and using antioxidants such as vitamins and minerals in hyperthyroid patients for diminishing the related side effects seem vital. The disturbed antioxidant system indicates the possible usefulness of supplementation with antioxidants to preventing the oxidative stress damage in hyperthyroid patients. The beneficial effects of anti-oxidants on oxidative stress induced by hyperthyroidism was shown in several animal models.^6^^-^^8^ On the other hand, use of herbal medicine for treatment of hyperthyroidism is well known in recent years due to their safety, high efficacy and lower cost.

**Fig. 1:**
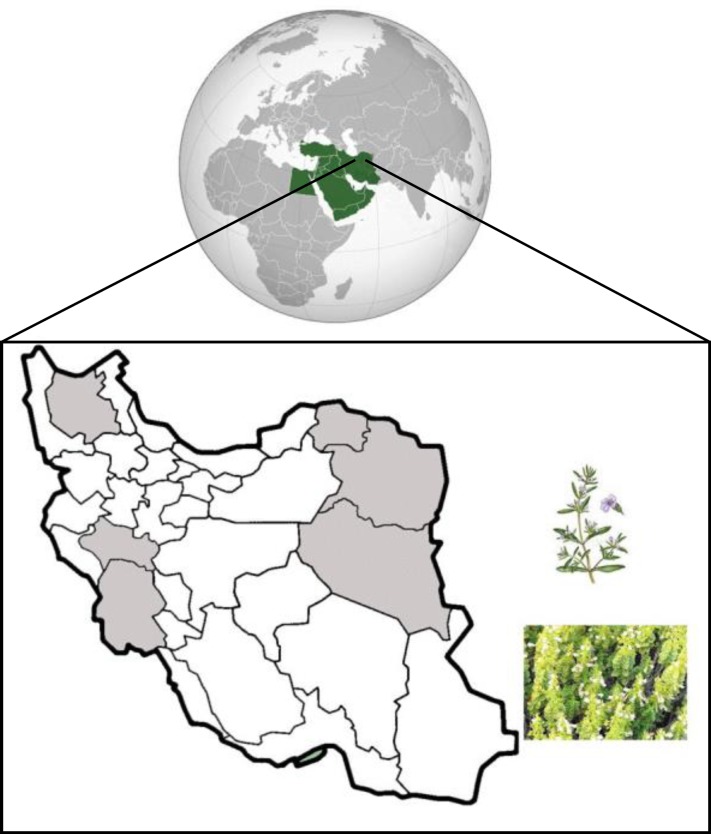
Geographical distribution of *Satureja khuzestanica *Jamzad in Iran. This plant is densely leafy, and broadly ovaiate-orbicular covered with white hairs

*Satureja khuzestanica* Jamzad (SKJ; Marzeh Khuzestani in Persian, family of Laminaceae) is an endemic plant of southern part of Iran. This plant is a subshrub, branched stem about 30 cm high, densely leafy, and broadly ovaiate-orbicular covered with white hairs. Geographical distribution and shape of this plant are shown in Figure 1. It is used as a folk medicinal plant, because of its therapeutic value as an analgesic and antiseptic properties.^9^ Active ingredients of *S. khuzestanica* essential oil (SKEO) are carvacol,^10^ antioxidant ^11^ and flavonoids with anti-oxidant and anti-thyroid properties.^12^ The SKEO has anti-inflammatory properties,^13^ ameliorates progression of diabetic nephropathy in uni-nephrectomized diabetic rats^14^ and improves inflammatory bowel disease by reduction of oxidative stress biomarkers.^15^ The extract also improves the reproductive potential of normal and cyclophosphamide treated male rats with enhancement of body antioxidant potency.^16^^,^^17^ However, a recent clinical trial in hyperlipidemic diabetic patients showed that administration of SKEO did not change blood oxidant levels.^18^ Therefore, the aims of this study were to evaluate the effect of SKEO alone or in combination with vitamin E on hyperthyroid lipid peroxidation (LP), glutathione peroxidase (GPx) activity and its so far unknown ameliorative effects on regulating hyperthyroidism in male rats as a human model. Vitamin E was selected as a potential antioxidant according to the reported effects based on our previous studies.^19^^,^^20^

## Materials and Methods

**Reagents. **Thiobarbituric acid, tert-butyl hydro-peroxide (t-BuOOH) and 1,1,3,3,-tetraethoxy propane (TEP) were purchased from Sigma Chemical Co. (St Louis, USA). Na_2_-NADPH was obtained from Fluka Chemical Co. (Buchs, St. Gallen, Switzerland). Hydrogen peroxide (H_2_O_2_) and sodium azide were obtained from Merck (Darmstadt, Germany). All other chemical reagents were of analytical grades and were supplied by commercial sources.

**Plant material and preparation of the essential oil. **The aerial parts of SKJ were collected during the spring season at the flowering stage of the plant from Khoarram-abad (Lorestan province, Iran) and the taxonomic identification of each plant was confirmed by botanist from Department of Botany of the Shiraz University, Shiraz, Iran. A voucher specimen (No. 58416) had been deposited previously at the National Herbarium of Iran, Research Institute of Forests and Rangelands. Preparation of essential oil was performed according to previous reports.^15^ Briefly, the collected parts of the plant were air-dried at ambient temperature in the shade and hydro-distilled using a Clevenger-type apparatus for 5 hr, giving yellow oil in 0.9% yield. The essence with density of 0.98 was dried over anhydrous sodium sulfate and stored at 4 ˚C.

**Animals and housing. **This work was done at the Laboratory Animal Center of Shiraz University of Medical Sciences and was approved by the Ethical Committee of the University. All efforts were made to minimize suffering during the experimental period. Forty male adult Sprague-Dawley rats weighing 225 ± 25 g and age of about 14 weeks obtained from the laboratory were used as mature rats in this study. The animals were housed in standard polypropylene cages with wire mesh top and maintained under conventional conditions with 12:12 hr light/dark cycle (lights on at 7:00 A.M.), at an ambient temperature of 22 ± 2 ˚C, and 50% relative humidity. The animals had free access to standard laboratory chow and tap water for 10 days before beginning the experimental procedure. Their cages were cleaned every third day and food/water were replenished.

**Experimental set-up. **The study comprised five different groups as shown in Table 1. The protocol of the study, including doses and duration of treatment, and groups were all designed according to previous studies.^6^^,^^17^ All animals were treated daily by gavage for 30 days. On the last day, rats were sacrificed by decapitation and blood samples were immediately collected into glass tubes without anticoagulant on ice and were allowed to clot. The blood samples were centrifuged at 700 *g* for 15 min to obtain serum. The clear non-hemolyzed supernatant sera were quickly removed and kept at –20 ˚C for further analysis.

**Biochemical analysis. **Serum analysis was carried out to measure total Thyroxine (T4) and tri-iodothyronine (T3), by radioimmunoassay (Beckman Coulter, Inc., Fullerton, USA) and TSH by ELISA kit (Cusabio Biotech Co., Newark, USA). The serum malondialdehyde (MDA) was assayed by a colorimetric method as described previously.^20^ The GPx activity of serum samples was measured by continuous monitoring of the regeneration of GSH from oxidized glutathione, GSSG, upon the action of glutathione reductase and NADPH according to the method of Fecondo and Augusteyn.^21^ The assay of GSH with DTNB was performed, followed by a standard Ellman’s method.^22^ Briefly, 0.5 mL (0.001 M) of 5,5-dithiobis,2-nitrobenzoic acid (DTNB) (Sigma Aldrich, Darmstadt, Germany) in a PBS was added to all sera and an absorbance of reaction product was observed after 5 min at 412 nm using UV-visible double beam spectro-photometer against a standard curve of GSH.

**Table 1:** Experimental set-up and grouping

**Group**	**Thyroid status**	**Interventions**		
		**Hyperthyroidism induction ** a	**Treatments**	
			**Vitamin E ** b	**SKEO ** c
**Negative control **	Euthyroid	-	-	-
**Positive control **	Hyperthyroid	+	-	-
**Trial I **	Hyperthyroid	+	+	-
**Trial II**	Hyperthyroid	+	-	+
**Trial III**	Hyperthyroid	+	+	+

a Hyperthyroidism induced by administering 0.0012% L-thyroxin, (Iran Hormone Co., Tehran, Iran) in drinking water for 30 days.

b Vitamin E used as dose as 200 mg kg^-1^ body weight.

c *Satureja khuzestanica* essential oil (SKEO) administered as dose as 225 mg kg^-1^ body weight.

**Statistical analysis. **All data are presented as mean ± standard error of mean (SEM) and analyzed using SPSS (Version 18; SPSS Inc., Chicago, USA) The data were analyzed analyzed by the Kolmogrov - Smirnov test. In this test all data had *p*0.05 and they were considered as parametric data. Therefore, they were subjected to a one way ANOVA followed by Tukey's test. Minimal statistical significance was accepted as *p* < 0.05.

## Results

Changes in serum concentrations of T_3_, T_4_, TSH, MDA, GSH and GPx activity in the different groups at the end of the experimental period are presented in Tables 2 and 3. A significant increase in serum T_3_ and T_4_ concentrations and decreased TSH value in all L-thyroxin treated rats were observed. On the other hand, in rats of trials I and III, the intensity of increasing in T_3_ value caused by L-thyroxin was lesser compared to other groups (Table 2). L-thyroxin treatment caused a significant increase in serum MDA concentration compared to positive control group in all treated rats and consumption of antioxidants alone or in combination restored the serum MDA level to the control one. The effect of SKEO and/or Vit. E on the GPx activity and GSH content of L-thyroxin treated rats are shown in Table 3. Although L-thyroxin treatment significantly reduced the GPx activity compared to control group, SKEO or Vit. E feeding (Trial I and II, respectively) restored GPx activity to the level of control group at the end of treatment. In addition, simultaneous use of SKEO and Vit. E (Trial III) increased the GPx activity significantly in comparison to all other groups. Also, GSH content showed significant decline in hyperthyroid against control group. About the GSH content, the most potent treatment was seen when the SKEO and Vit. E used simultaneously. They could significantly compensate the decreasing of GSH in comparison to control group (Table 3).

**Table 2:** Serum total tri-iodothyronine (T3), thyroxine (T4), and thyroid-stimulating hormone (TSH) concentration (Mean ± SEM) in different groups after 30 days treatment (n = 8).

**Groups**	**T**_4_ ** (nmol** **L**^-1^**)**	**T**_3_ ** (nmol** **L**^-1^**)**	**TSH ** **(µIU mL** ^-1^ **)**
**Negative control **	3.25 ± 0.40^a^	49.00 ± 14.56^a^	2.73 ± 1.08^a^
**Positive control **	12.34 ± 0.84^b^	226.00 ± 69.12^b^	0.07 ± 0.07^b^
**Trial I **	14.24 ± 1.51^b^	249.00 ± 78.51^b^	ND*
**Trial II**	12.32 ± 0.88^b^	107.00 ± 38.87^c^	0.61 ± 0.49^b^
**Trial III**	12.91 ± 1.82^b^	109.00 ± 55.01^c^	0.09 ± 0.09^b^

abc Different superscript letters in each column indicate significant differences (*p *< 0.05).

* ND: Not detectable.

**Table 3:** Serum malondialdehyde (MDA) and reduced glutathione (GSH) concentration with serum glutathione peroxidase (GPx) activity in different groups after 30 days treatment. Data are presented as Mean ± SEM (n = 8).

**Groups**	**MDA (nmol mL** ^-1^ **)**	**GPx (U mL** ^-1^ **)**	**GSH (nmol mL** ^-1^ **)**
**Negative control **	1.22 ± 0.25^a^	131.38 ± 2.25^a^	5.16 ± 0.39^a^
**Positive control **	2.80 ± 0.28^b^	102.18 ± 3.91^b^	3.30 ± 0.15^bc^
**Trial I **	1.24 ± 0.11^a^	133.33 ± 5.80^a^	4.45 ± 0.28^ad^
**Trial II**	1.12 ± 0.16^a^	128.07 ± 3.09^a^	4.08 ± 0.08bde
**Trial III**	1.17 ± 0.30^a^	156.75 ± 3.55^c^	5.01 ± 0.42^ae^

**abcde:** Different superscript letters in each column indicate significant differences (*p* < 0.05).

## Discussion

This study provides the assessment of the MDA and GSH levels and also GPx activity changes in hyperthyroid rat in response to SKEO and/or Vit. E consumption. Hyperthyroidism was confirmed by the significant increase of T_4_ and T_3_ concentration and the decreased level of TSH in all L-thyroxin treated rats. However, in SKEO and SKEO + Vit. E treated rats, the extent of increasing T_3_ value caused by L-thyroxin was lesser (about 60%) compared to other hyperthyroid animals. In these groups, serum T_3_ concentration was reduced without changes in T_4_ level. The T_4_ acts as the main source of T_3_ production and 87.0% of circulating T_3_, which produced mostly in the liver, is result of T_4_ to T_3_ conversion by 5' deiodinase enzyme. Flavonoids are potent non-toxic 5' deiodinase inhibitors in microsomal membrane and intact rat hepatocytes.^23^ The SKEO contains several flavonoids and can decrease T_3_ through inhibiting 5' deiodinase activity due to this mechanism.

In the current study, SKEO and/or Vit. E supplementation normalized elevated serum MDA, as a LP indicator, in hyperthyroid rats. Our finding about antioxidant properties of *S. khuzestanica *Jamzad are in agreement with all other previously researches that reported the antioxidant and anti lipid peroxidation activities of this traditional plant.^18^^,^^24^^,^^25^ In fact, LP is the automatic chain reactions that produce free radicals in cell membranes and MDA is the most important reactive aldehyde metabolite of free radicals that was produced through LP.^6^ Hyperthyroidism could induce superoxide generation followed by hydroxyl radicals at the site of ubiquinones that immediately produce free radical-mediated LP, and increases MDA concentration.^26^ Our data showed that T_3_ level in SKEO gavage feeding hyperthyroid animals was markedly decreased suggesting that LP may reduce partly, due to its T_3_ level reduction. Carvacrol, the major components of SKEO, acts as a strong antioxidant and can improve the condition of oxidative stress through inhibition of xanthine oxidase, chelation of transition metals, scavenging free radicals, blocking of oxidative reactions and reinforcing of cellular antioxidant capacity.^27^ Hence, SKEO may reduce LP through carvacrol antioxidant property. Cyclic adenosine monophosphate (cAMP) is a second messenger that controls many key cellular functions. It can diminish oxidative stress in many biological systems and diseases.^28^ Both cAMP and cGMP elevation that suppress TNFα secretion from peripheral monocytes, macrophages and T cells, can inhibit the production of superoxide anions from neutrophils.^29^ The only way to inactivate cAMP is to degrade it through the action of cAMP phosphodiesterases (PDEs), a class of enzymes that be able to cleave the phosphodiester bonds in either cAMP or cGMP in order to yield 5’-AMP and 5’-GMP, respectively. *Satureja* species contain flavonoids that have PDE inhibitory effects an therefore, SKEO may reduce LP due to its PDE inhibition.^30^

We demonstrated that SKEO or Vit. E gavage feeding increased GPx activity. Combination therapy of both antioxidants could significantly increase the GPx activity compared to the administration of each by itself. SKEO treatment in hyperthyroidism may decrease the workload of GPx and thereby maintaining its activity through the major constituents, carvacrol and flavonoids. Carvacrol scavenging superoxide radicals and hydrogen peroxide with reduction the free-radical-mediated inactivation of enzyme.^31^ On the other hand, flavonoids are able to donate hydrogen atoms to tocopheryl radicals and therefore recycle antioxidant, like α-tocopherol.^32^ Thus, a combination of SKEO and Vit. E may be more effective in attenuating oxidative stress induced by hyperthyroidism than either antioxidant used alone. 

To the best of our knowledge, this is the first report on the antioxidant and antithyroid activities of *S. khuzestanica *Jamzad in hyperthyroid rat. In conclusion, we have described the usefulness of this plant in attenuating LP in the hyperthyroid rats which in turn may potentially beneficial in hyperthyroid patients. Further pharmacological and toxicological study may be warranted to establish any potential therapeutic use of this plant and its active compounds. 
